# Supplementary material for: Functional reduction in pollination through herbivore-induced pollinator limitation and its potential in mutualist communities
Source: Nat Commun. 2017 Dec 11;8:2031. doi: 10.1038/s41467-017-02072-4 (PMC5725495; doi:10.1038/s41467-017-02072-4)
Supplement: Supplementary file 1 — Supplementary information [file 41467_2017_2072_MOESM1_ESM.pdf]

Supplementary Note 1 – Supplementary Notes on Curve Fitting Against Raw Data

In this work, two data sets were used from studies which investigated the effects of herbivory on pollinator visitation across a continuous spectrum of herbivory: Kessler et al 2011 and Barber et al 2012[1, 2]. While a number of studies have found evidence of herbivory reducing the amount of pollination individual plants receive, those studies often use categorical treatments of pollination measured with and without herbivore damage[3, 4, 5]. Few have studied pollination across on a continuous spectrum of herbivore damage as was done in these two studies. This data was used to curve fit and find the best possible support for the form of functional response of pollinator visitation rates to different levels of herbivory. As written in the main paper, the functional response is labeled $v(c, h)$ where h is the percentage of herbivore damaged leaves and c is parameter which describes the intensity of the effect of h.

Statistics on the raw data from each study can be found below. Each data was fitted against 6 models.: 1.) Type I or linear decline response, 2.) Type II declining response, 3.) Type III declining response, 4.) Mixed Saturating decline, 5.) Concave declining function, 6.) a generalized Poisson fit. Type I, II, and III functional responses are named as such due to their dynamic similarity to functional responses seen in predation and mutualistic interactions. The Mixed Saturating model tests the effect of a response model with a scalar multiplier on h and a potential non-integer exponent (see Supplementary Table 1). The concave function allows for the testing of a potential threshold effect. These response models were chosen based on their established use in the theoretical literature, their shown applicability in other interactions (such as predation and mutualist interaction), and their ability to cover potential dynamic responses to herbivory. Finally, the Poisson fit allows us to compare the functional response to a more traditional test of this type of count data. Models were fit to the data in the statistical software R and compared using AICc weights given their nonlinearity.

Fitting raw data from Kessler et al 2011

Experiments in Kessler et al 2011 were conducted in Peru. This field study measured the proportion of flowers with pollination marks as a proxy for pollinator visitation and as a function of herbivore damage in the wild tomato *Solanum peruvianum*. Pollination marks were measured in relation to herbivore leaf damage experienced by individual *S. peruvianum* plants. *S. peruvianum* is attacked by a diverse set of herbivorous insects and pollinated by specialist bees in the *Apidae*, *Colletidae*, and *Halictidae* families. For more information, please see the original paper[1].

The results of curve fitting the 6 candidate models to Kessler's raw data are displayed in Supplementary Table 1. This shows no entirely definitive support for a single model. The Linear, Type II, and Mixed Saturating models are all shown to have some comparable support. However, as with the results shown in the main paper, the Type II response has the highest support and the Mixed Saturating

Model has a very similar form to the Type II ($b = 1.196$). We also note that the shape of the Poisson predicted fit does mimic the exponential decay relationship modeled by the Type II functional response (Supplementary Figure 1). While the level of support in the raw data for the Type II form is more limited, the above reasoning and the results described in the main paper lead us to argue that the Type II response is the best suited functional response form from this data set. It should also be noted that not allowing the y-intercept to vary and fixing it to 100% increases the AICc weight of the Type II functional response to 0.85 and 0.62 in the averaged data fit and raw data fit respectively.

Supplementary Table 1 | Curve fitting results from Kessler et al 2011 raw data.

Model fitting to original data from *Solanum peruvianum* field experiments in Peru. Curve fittings of six candidate response models to Kessler et al 2011 raw data[1]: Type I/Linear, Type II, Type III, Mixed Saturating, Concave, Poisson fit. Here h represents the level of herbivory. The parameters c and b determine the shape of the curve and i is the intercept. Estimated parameters that are significant have their p values bolded. The Type II functional response has the highest Akaike Information Criterion weight of 0.46021.

Fitted models to real data w/ intercepts	Estimated Parameters	Significance and Fit	AICc	AICc weight
1.)Linear: $\sim ch + i$	$c = -0.64$, $i = 0.8583$	$p = (c)\mathbf{6.98e^{-13}}$ $(i)\mathbf{2e^{-16}}$ $R^2 = .2049$	168.3532	0.21497
2.)Type II: $\sim \frac{i}{1 + ch}$	$c = 1.7577$, $i = 0.9440$	$p = (c)\mathbf{7.3e^{-7}}$ $(i)\mathbf{2e^{-16}}$	166.8308	**0.46021**
3.)Type III: $\sim \frac{i}{1 + ch^2}$	$c = 2.3514$ $i = 0.8282$	$p = (c)\mathbf{6.1e^{-6}}$ $(i)\mathbf{2\ e^{-16}}$	169.8738	0.10050
4.)Mixed Saturating: $\sim \frac{i}{1 + ch^b}$	$c = 1.8948$ $b = 1.1964$ $i = 0.9164$	$p = (c)\mathbf{1.68e^{-5}}$ $(b)\mathbf{.000163}$ $(i)\mathbf{2e^{-16}}$	168.3841	0.21167
5.)Concave: $\sim i * \left(1 - \frac{h}{100}\right)^c$	$c = 0.6062$, $i = 0.8396$	$p = (c)\mathbf{4.52e^{-9}}$ $(i)\mathbf{2e^{-16}}$	174.0195	0.01265
6.) Poisson fit on count data:	Intercept$= 0.9190$ $\beta = -0.9983$	$p =$(Intercept)0.154 $(\beta)\mathbf{1.43e^{-12}}$	798.8534	$2.6362\ e^{-138}$

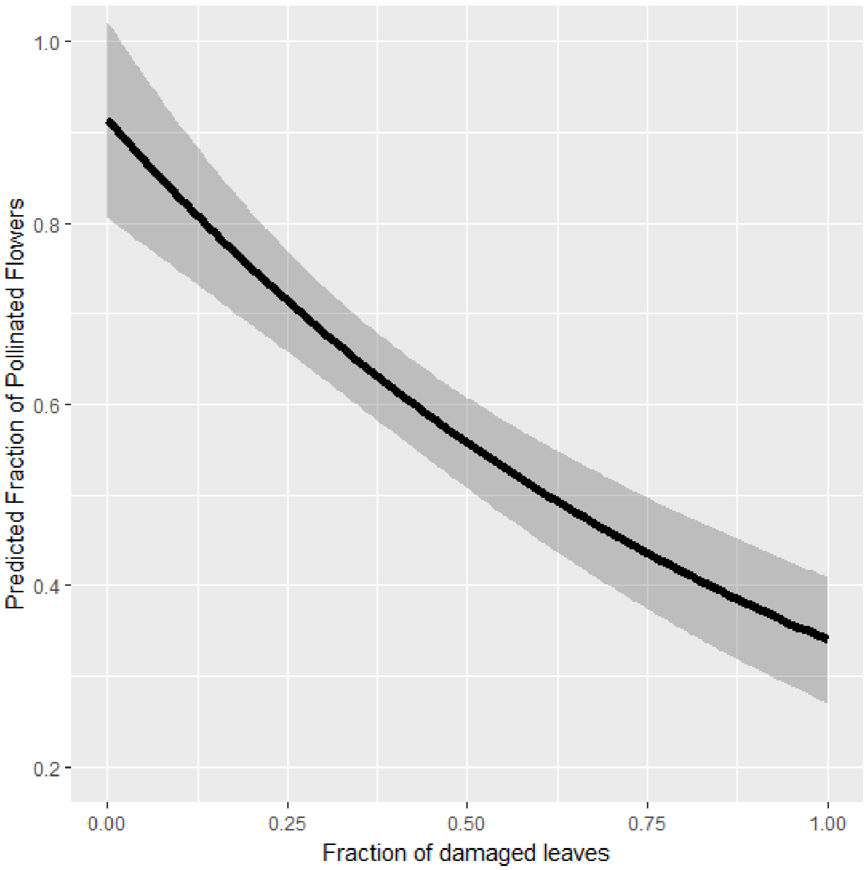

Supplementary Figure 1 | Plotted fit of the Poisson fit.
Plotting the predicted relationship between herbivore damage and pollinator visited flowers derived from the generalized Poisson fit using the raw count data.

Fitting raw data from Barber et al 2012

The flowering plant used in this was *Cucumis sativus* (cucumber, *Cucurbitaceae*), a widely cultivated annual, monoecious herb reliant on pollinators to vector pollen between male and female flowers. *Cucumis sativus* is pollinated by a numerous insects, including generalist bees (honey bees and bumble bees), a variety of solitary bees, butterflies, and hover-flies (*Syrphidae*). The herbivorous insect of interest was *Acalymma vittatum*, a common specialist herbivore and agricultural pest of *Cucurbitacea* in the northeast United States. Herbivory occurs by adults feeding on stems and leaves above as well as larvae eating roots below ground. At various levels of herbivory, per replicate plant, pollinator visits were recorded. For more information, please see the original paper[2]. In this data set, all pollinators listed above are grouped together in observations. Data for just honey bees and bumble bees was also tested separately, but is not displayed here. This data and the six fitted models are displayed in Supplementary Figure 2. Data was not averaged in this case as the coverage across leaf damage percent was not even across the full spectrum.

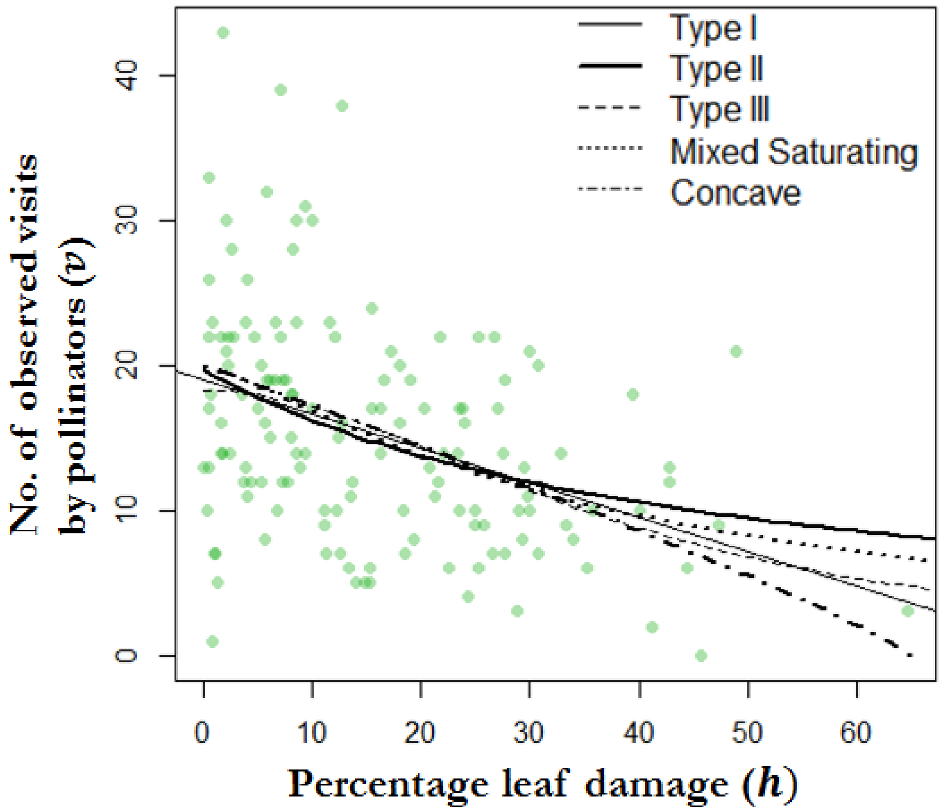

Supplementary Figure 2 | Fits of candidate models to Barber et al 2012 data.
Effects of herbivore damage (h) on number of observed visits by pollinators (v) on *Cucumis sativus*. Individual data points are shown as green dots. While there is a significant negative effect of herbivory on pollinator visits, none of the five candidate models are shown to have noticeably better fits than any others. The fits of five of the candidate models are overlaid over the data as different lines. The legend describes which line represents which model. The lack of any singular best fit model is reflected in the fact all five models overlap a great deal.

The results of curve fitting the six candidate models to Barber's data are displayed in Supplementary Table 2. While the negative effect of increased levels of herbivory is significant[2], this data set shows no support for any one out of the six candidate models tested here. The AICc weights are particularly even across all six models. Given that the bulk of the pollinators observed in this study are large generalists (honey bees and bumble bees), it is reasonable to expect different results than those garnered from Kessler et al's 2011 paper, where the bees were smaller specialists[1]. While there are numerous speculative reasons for these differences, what this result shows is that the form of $v(c, H)$ is likely different across species and systems. This may be an important component when considering interactions among multiple pollinators on shared resource flowers.

Supplementary Table 2 | Curve fitting results from Barber et al 2012 data.

Model fitting to original data from *Cucumis sativus* field experiments from Barber et al 2012[2]. Curve fittings of six candidate response models: Type I/Linear, Type II, Type III, Mixed Saturating, Concave, and Poisson. Here *h* represents the level of herbivory. The parameters *c* and *b* determine the shape of the curve and *i* is the intercept. Estimated parameters that are significant have their p values bolded. While the Type III functional response has the highest Akaike Information Criterion weight of 0.2726, no one model has the clear advantage.

Fitted models to real data w/ intercepts	Estimated Parameters	Significance and Fit	AICc	AICc weight
1.)Linear: $\sim ch + i$	$c = -0.2355$<br>$i = 18.967$	$p = (c)\mathbf{1.82e^{-6}}$<br>$(i)\mathbf{2e^{-16}}$,<br>$R^2 = 0.1465$	956.349	0.2436
2.)Type II: $\sim \frac{i}{1 + ch}$	$c = 0.02175$<br>$i = 19.7184$	$p = (c)\mathbf{0.001}$<br>$(i)\mathbf{2e^{-16}}$	957.073	0.1696
3.)Type III: $\sim \frac{i}{1 + ch^2}$	$c = 6.752e10^{-4}$<br>$i = 18.27$	$p = (c)\mathbf{0.00118}$<br>$(i)\mathbf{2\ e^{-16}}$	956.125	0.2726
4.)Mixed Nonlinear: $\sim \frac{i}{1 + ch^b}$	$c = 0.00315$<br>$b = 1.561$<br>$i = 18.7612$	$p = (c)0.6716$<br>$(b)\mathbf{0.0204}$<br>$(i)\mathbf{2e^{-16}}$	957.699	0.1241
5.)Concave: $\sim i * \left(1 - \frac{h}{65}\right)^c$	$c = 0.7377$<br>$i = 18.844$	$p = (c)\mathbf{6.41e^{-5}}$<br>$(i)\mathbf{2e^{-16}}$	956.847	0.190
6.) Poisson fit on count data:	Intercept= 19.5275<br>$\beta = -0.0171$	$p =$(Intercept)$\mathbf{2e^{-16}}$<br>$(\beta)\mathbf{2e^{-16}}$	1097.812	$4.66e^{-32}$

Supplementary Note 2 – Fitting binned data from Kessler et al 2011 with weighted bins

In using the binned data, it is reasonable to also consider the effects of standard errors of the mean values from the bins in estimation and comparison of the curves. This can be done by counting the number of observations per bin and using them as weights with the "weights" argument provided in the nls function in R. When we do so, we see no change in the main result, the Type II functional form is still the best supported model from the analysis (Supplementary Table 3). However, there is more comparable support for the Type I/Linear form and still appreciable support for the Mixed Saturating form. These

results provided additional prompting to study the effects of the other functional forms on the model results (see Supplementary Note 8 – Supplementary Note 11).

Supplementary Table 3 | Curve fitting results considering bin weights.

Table describing the results of the curve fitting to the 5 candidate response models: Type I/Linear, Type II, Type III, Mixed Saturating, Concave when including the weights in each bin. Here h represents the level of herbivory. The parameters c and b determine the shape of the curve and i is the intercept. Equation representations of each model are given along with a pictorial example of each model. Estimated parameters that are significant have their p values bolded. The Type II functional response has the highest Akaike Information Criterion weight of 0.587.

Fitted models to averages	Example of functional form	Estimated Parameters	Significance and Fit	AICc	AICc weight
1.)Type I/Linear: $\sim ch + i$	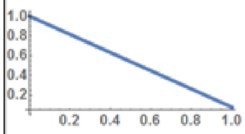	$c = -0.654$, $i = 0.864$	$p = (c)\mathbf{4.13e^{-6}}$ $(i)\mathbf{8.43e^{-10}}$ $R^2 = 0.911$	-315.09	0.215
2.)Type II: $\sim \frac{i}{1+ch}$	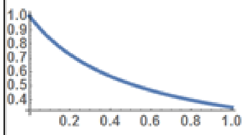	$c = 1.807$, $i = 0.953$	$p = (c)\mathbf{2.77e^{-5}}$ $(i)\mathbf{1.89e^{-9}}$	-317.10	**<u>0.587</u>**
3.)Type III: $\sim \frac{i}{1+ch^2}$	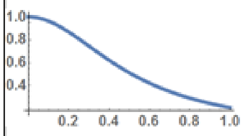	$c = 2.491$ $i = 0.838$	$p = (c)\mathbf{3.57e^{-4}}$ $(i)\mathbf{2.86e^{-9}}$	-312.59	0.062
4.)Mixed Saturating: $\sim \frac{i}{1+ch^b}$	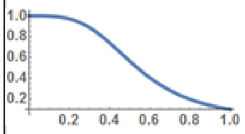	$c = 2.000$ $b = 1.243$ $i = 0.920$	$p = (c)\mathbf{1.13e^{-4}}$ $(b)\mathbf{0.242e^{-4}}$ $(i)\mathbf{2.62e^{-8}}$	-313.90	0.119
5.)Concave: $\sim i * \left(1 - \frac{h}{100}\right)^c$	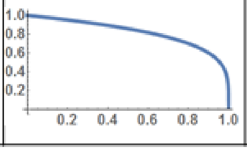	$c = 0.606$, $i = 0.842$	$p = (c)\mathbf{2.58e^{-4}}$ $(i)\mathbf{2.36e^{-8}}$	-310.16	0.018

Supplementary Note 3 - Obligate mutualism without functional HIPL

The full effect of $v(c, H)$on system persistence is made clear by first setting $c = 0$, making $v(c, H) = 1$. This effectively eliminates the mechanism of herbivore-induced visitation reduction from the model. Doing this also allows us to quickly verify previous theoretical work and show the fragile nature of antagonized mutualisms in their most basic theoretical formulation. The categories of possible

dynamics are relatively short. The system can be sustained by both stable equilibria and stable limit cycles. However, the two different dynamics of system persistence are mutually exclusive across parameter space such that no parameter combination creates a phase space with both a stable equilibrium and a stable limit cycle. Antagonized obligate-mutualisms function dynamically similar to a predator-prey system with the prey split into two mutually dependent populations. Classic Lotka-Volterra predator-prey systems also result in only one dynamic category of persistence per parameter combination. Therefore, the lack of overlapping in dynamical categories of persistence has reasonable precedence in this and other models.

The range of parameter space which creates dynamics that support persistence is quite narrow. Supplementary Figure 3 shows the results of a Jacobian stability analysis across $\{b_F, b_P, r_H\}$ parameter space. The results of the stability analysis are visualized as colors across parameter space. Parameter combinations which create locally stable equilibria are shown in green, while the blue represents space where equilibria are unstable and result in system extinction. The actual equilibrium values are written out parametrically in S1-S3. The slim orange space represents the space which creates unstable equilibria but stable limit cycles (sustained oscillations). The parameter space which supports limit cycles had to be compiled in a list of data because the surface was too thin for the available software, Mathematica, to render.

$$\boldsymbol{F}^* \rightarrow \frac{d_H}{c_{FH} r_H - d_H h_H} \tag{S1}$$

$$\boldsymbol{H}^* \rightarrow c_{FH}\left(\frac{-a d_H + d_F d_H h_H - c_{FH} d_F r_H}{(d_H h_H - c_{FH} r_H)^2} + \frac{b_F d_P v}{a d_H h_H - a d_H h_P - a c_{FH} r_H} + \frac{b_F b_P d_H v^2}{a(d_H(h_P - h_H) + c_{FH} r_H)^2}\right) \tag{S2}$$

$$\boldsymbol{P}^* \rightarrow \frac{-d_P + \left(\frac{b_P d_H v}{d_H(h_P - h_H) + c_{FH} r_H}\right)}{a} \tag{S3}$$

It is readily apparent that, in this simple form, the system can only potentially persist at very low levels of herbivory (Supplementary Figure 3). Values of both b_P and b_F have minimal effects on the mutualism's ability to persist across different levels of herbivory (i.e. different values of r_H). Overall, this simple version of the system has a narrow range of parameter space where all three variables can coexist without going extinct due to herbivory.

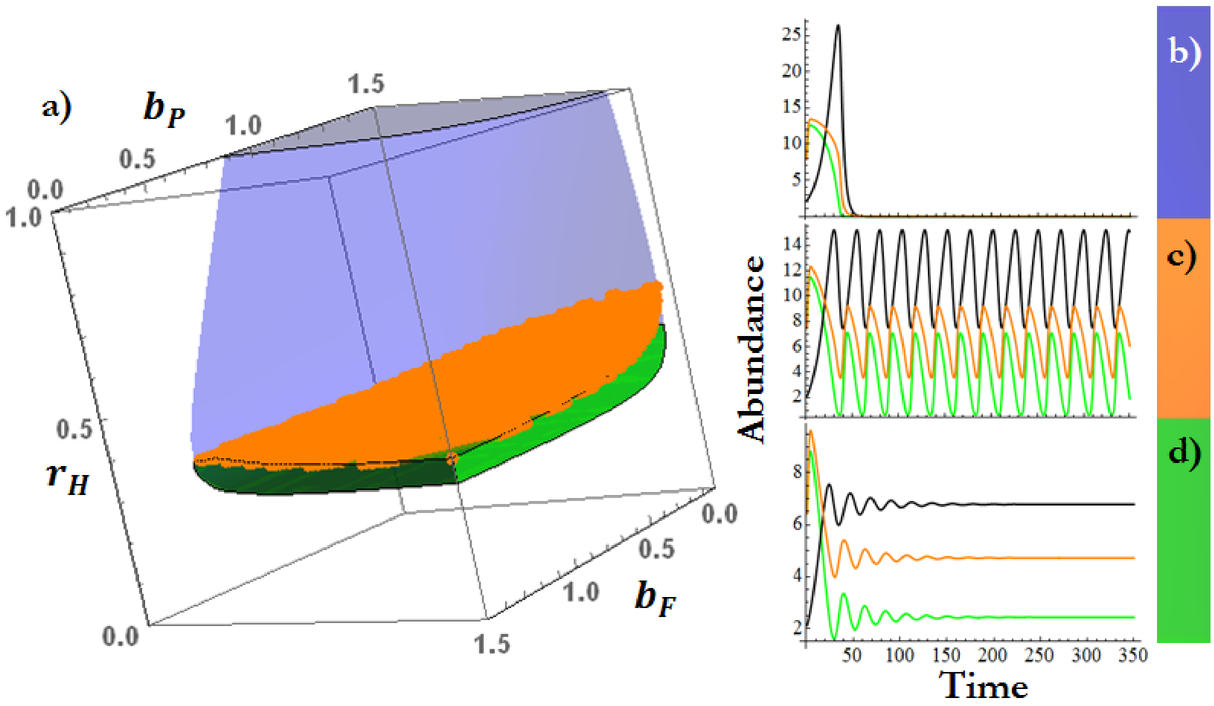

Supplementary Figure 3 | Dynamics across parameter space for Equation 1 without HIPL. Exploration of the different type of model behaviors/dynamics across different parameter values in the obligate model with no HIPL ($r_F = 0, c = 0$). a) A representation of different dominant asymptotic dynamics across $\{b_F, b_P, r_H\}$ parameter space. Parameter b_F represents the reproductive benefit of pollination to the flowering plant population. Parameter b_P represents the reproductive benefit of pollination to the pollinator population. Parameter r_H represents the attack rate of the herbivore. The green space represents parameter combinations where the non-zero equilibrium is locally stable. The thin orange space represents combinations where the non-zero equilibrium is unstable but a stable limit cycle exists. The parameter space which supports limit cycles had to be compiled in a list of data because the surface was too thin for the available software, Mathematica, to render. Both limit cycles and stable equilibria represent system persistence. The blue space represents space where the non-zero equilibrium exists, but is unstable resulting in the system going extinct. Supplementary Fig 3b-3d represent example simulations/time series from each different parameter grouping in Supplementary Fig 3a. In the time series, green lines represent F, orange lines represent P, and black lines represent H. b) An example simulation from the extinction producing region of parameter space (blue region in Supplementary Fig 3a). c) An example simulation from the limit cycle producing region of parameter space (orange region in Supplementary Fig 3a). c) An example simulation from the stable equilibrium producing region of parameter space (green region in Supplementary Fig 3a). The other parameter values are as follows: $d_H = .25$, $d_P = d_F = .2$, $r_F = 0$, $c_{FH} = 1$, $c = 0$, $h_H = h_P = 1$. See Table 1 in the main text for parameter and variable definitions.

In addition to the limited amount of parameter space which creates persistent systems, systems which do have a potential dynamic of system persistence (either equilibrium or limit cycle) can be perturbed into phase space which leads to system collapse. This can happen when trajectories are moved out of the basin of attraction of either the stable equilibrium or limit cycle and into the basin of attraction of the 0-equilbrium absorbing state in phase space. An example of this is given in Supplementary Figure 4. For more detailed descriptions of the basin of attraction see Strogatz 1994[6]. Supplementary Figure 4 shows that the basin of attraction of the stable equilibrium is limited, mainly in the H direction, such that higher H values means trajectories are caught in the basin of the 0-equilbrium and the communities goes extinct. This exercise corroborates past work which describes the seemingly fragile nature of these systems under base model formulation.

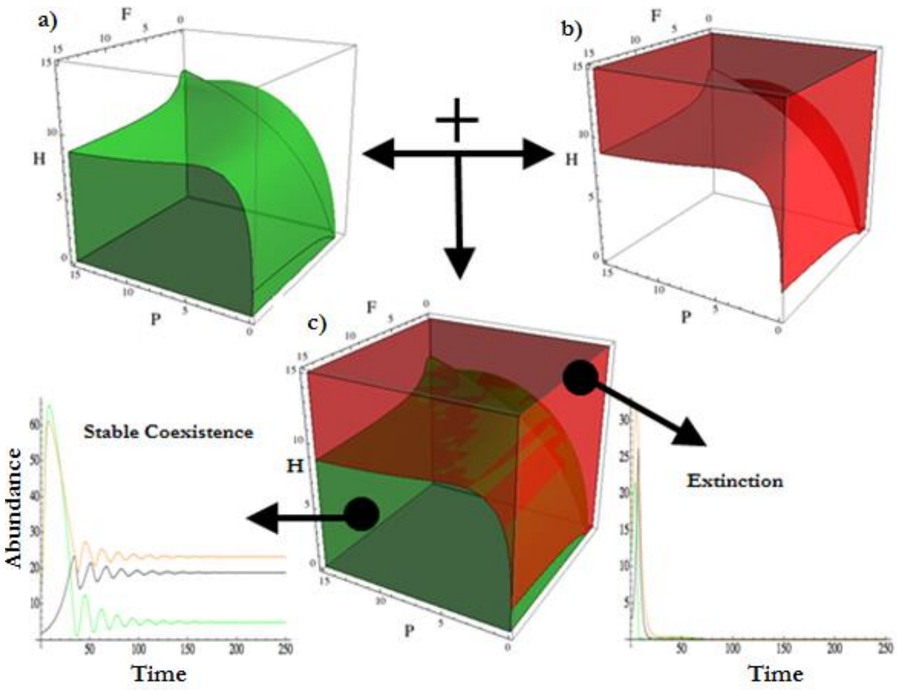

Supplementary Figure 4 | Basins of attraction for Equilibrium 4 and the 0-equilibrium. Examples of basins of attraction in $\{F, H, P\}$ phase space and their resulting model behaviors. F-flowering plant population, H-herbivore population, P-pollinator population. a) The basin of attraction for a stable equilibrium where $F^*, H^*, P^* > 0$ shown in green. All initial conditions inside this basin lead to stable equilibria. b) The basin of attraction for the 0-equilbrium, (0,0,0), shown in red. All initial conditions in this basin lead to extinction. c) Combining a) and b) shows that the basins completely fill the phase space. Asymptotic model behavior is shown to depend on which basin of attraction initial conditions start in. When initial conditions start in the green (basin of attraction of the stable equilibrium), trajectories

experience dampened oscillations and the system persists in stable coexistence. However, when initial conditions start in the red (basin of attraction of the (0,0,0) state, trajectories are pulled to extinction due to saturation with herbivores. In the time series, green lines represent F, orange lines represent P, and black lines represent H. See Table 1 in the main text for parameter and variable definitions.

Supplementary Note 4 - Obligate mutualism with functional HIPL

This section studies Equation 1 when $r_F = 0$ and $c > 0$. The expression for F^* is written below. Parametric expression for H^* and P^* are too large for print. Please use the following Mathematica code to examine the equilibria.

$$\text{FHPSol} = \text{Solve}[F * ((\text{bf}/(1 + c * H)) * (P/(1 + hp * F)) - a * F) - (\text{rh} * F * H)/(1 + hh * F) - \text{df} * F == 0 \&\& cfh * \text{rh} * F * H/(1 + hh * F) - \text{dh} * H == 0 \&\& P * ((\text{bp}/(1 + c * H)) * (F/(1 + hp * F)) - a * P) - \text{dp} * P == 0, \{F, H, P\}];$$

The addition of HIPL expands the range of herbivory levels that the mutualism can withstand. The mechanism of this expansion and increased resilience comes from the asynchronous oscillations of herbivore populations ($H(t)$) and $v(c, H) = \frac{1}{1+cH}$ through time (Supplementary Figure 5). When $c > 0$ increased herbivore abundance dynamically lowers the interaction rate between flowers and pollinators. While this does obviously reduce the population growth of both the flowering plant and the pollinator, this indirectly lowers $\frac{dH(t)}{dt}$ and causes the herbivore population growth to slow so that it never saturates the system as it did when $c = 0$. The greater the value of c, the quicker high H abundance lowers visitation rates and consequently, the more controlled the herbivore population (Figure 3).

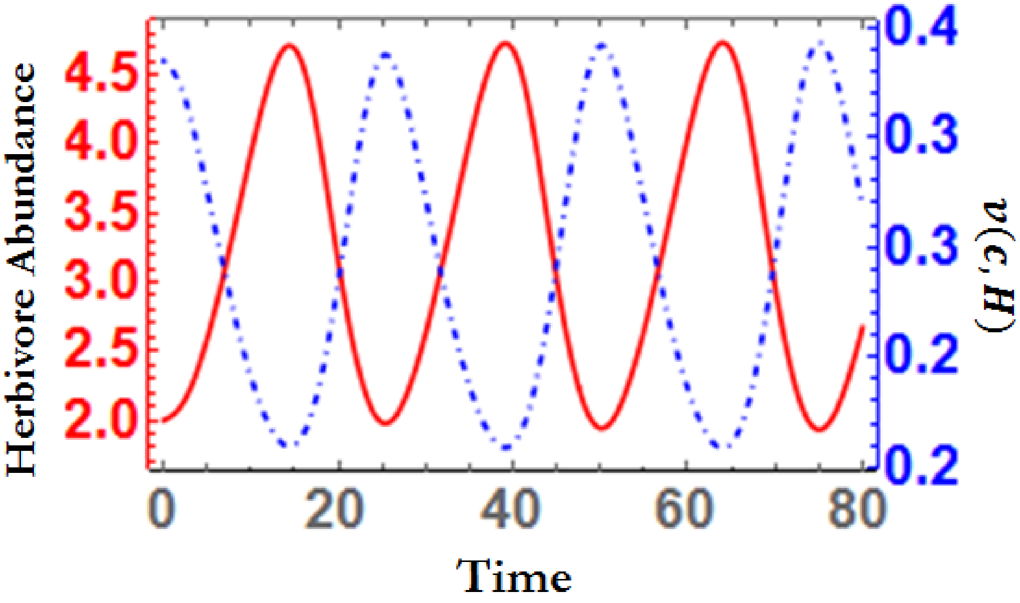

Supplementary Figure 5 | Dynamics of herbivore abundance and v across time.

Asynchronous oscillations of herbivore abundance (red, left axis) and pollinator visitation rates (blue/dashed, right axis). These asynchronous oscillations come from the form of $v(c,H)=\frac{1}{1+cH}$.

Analysis of this version of the model started with analysis of available equilibria and their stability. Unfortunately, the seemingly modest addition of function $v(c,H)$ to the model creates analytically incalculable equilibria. Simple algebraic manipulation of $\frac{dH}{dt}$ can show that: $F^*=\frac{d_H}{c_{FH}r_H-d_H h_H}$.

On the other hand, both P^* and H^* cannot be written completely parametrically in a length that would fit within reasonably sized manuscript. Therefore, using the Jacobian and eigenvalues to identify all general relationships between parameter values and stability in the model was not feasible. However, it is possible to find an inverse relationship that exists between the values of the equilibria of P^* and H^*. Starting with $\frac{dP}{dt}=0$ it is possible to show that:

$$\frac{dP}{dt}=P^*\left(\frac{b_P v(c)F^*}{1+h_P F^*}-\alpha_P P^*\right)-d_P P^*=0 \tag{S4}$$

$$P^*\left(\frac{b_P v(c)F^*}{1+h_P F^*}-\alpha_P P^*\right)=d_p P^* \tag{S5}$$

substitute: $$F^*=\frac{d_H}{(c_{FH}r_H-d_H h_H)}\rightarrow P^*\left(\frac{b_P v(c)\frac{d_H}{(c_{FH}r_H-d_H h_H)}}{1+\frac{h_P d_H}{(c_{FH}r_H-d_H h_H)}}-\alpha_P P^*\right)=d_p P^* \tag{S6}$$

$$\left(\frac{b_P v(c)\frac{d_H}{(c_{FH}r_H-d_H h_H)}}{\frac{(c_{FH}r_H-d_H h_H)+h_P d_H}{c_{FH}r_H-d_H h_H}}-\alpha_P P^*\right)=d_p \tag{S7}$$

$$\left(b_P v(c)\frac{d_H}{(c_{FH}r_H-d_H h_H)}\left(\frac{c_{FH}r_H-d_H h_H}{(c_{FH}r_H-d_H h_H)+h_P d_H}\right)-d_p\right)=\alpha_P P^* \tag{S8}$$

$$\frac{1}{\alpha_p}\left(\frac{b_p v(c)d_H}{(c_{FH}r_H-d_H h_H)+h_P d_H}-d_p\right)=P^* \tag{S9}$$

Since $v(c)=\frac{1}{1+cH}$ it is clear to see that $P^*\sim\frac{1}{H^*}$ (S9). While this is an intuitive result, the lack of full parametric expressions of equilibria means numeric approaches must be taken to understand the effect of changing model parameters. This was done using 2-dimensional bifurcation heatmaps for H and P respectively as was shown in Figure 4 for F (Supplementary Figure 6). The expanded resilience to higher values of r_H as c increases is clear. Despite c representing a necessary decline in interaction between the

mutualists, it is actually the herbivore population which has the most apparent population decline with higher values of c.

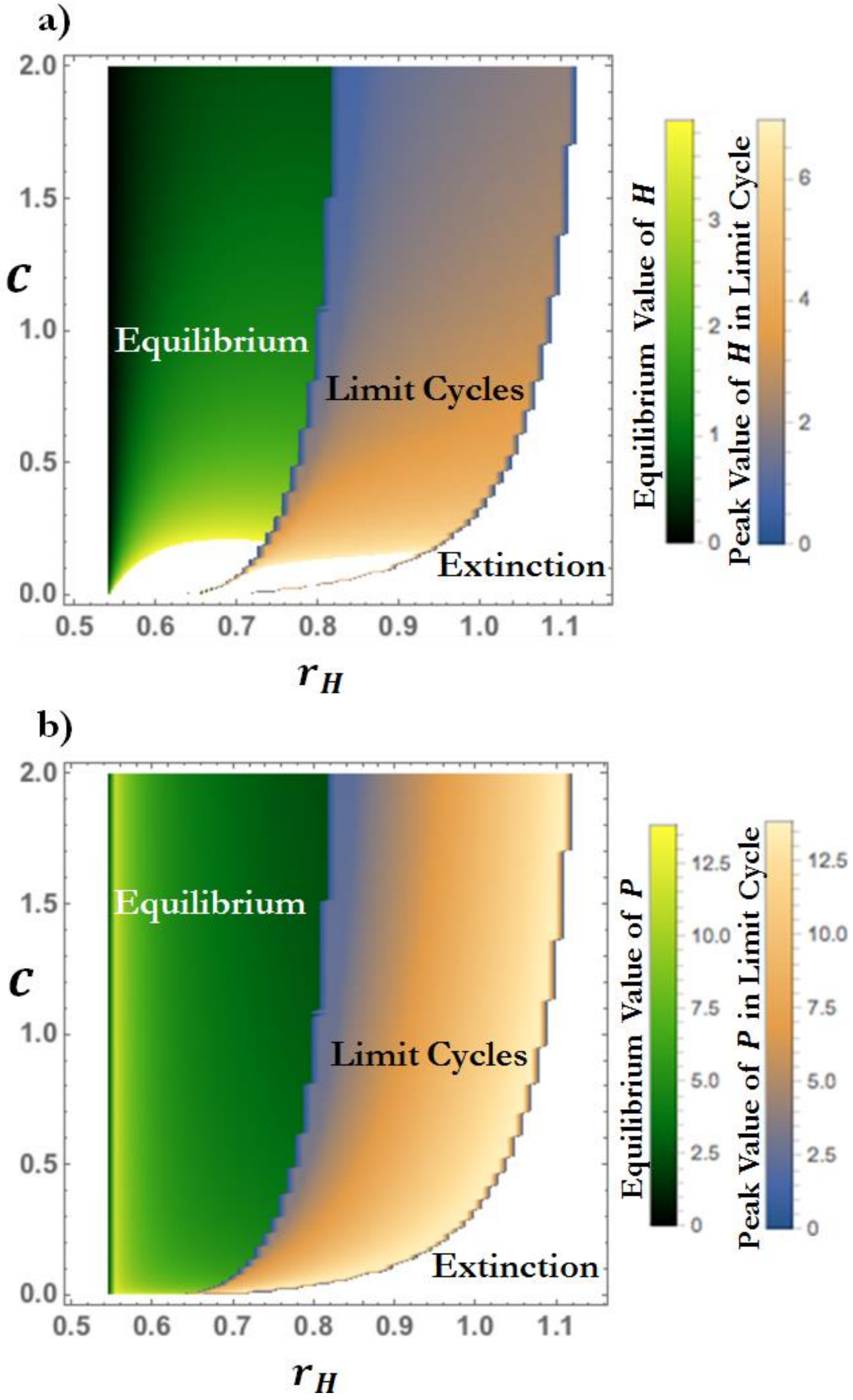

Supplementary Figure 6 | 2-D heatmaps for H and P in obligate mutualism community. 2-dimensional bifurcation heatmap showing the abundance for a) H (herbivore population) and b) P (pollinator population) across values for parameters c and r_H in the asymptotic behavior of the model. Parameter c represents the degree of herbivore-induced pollinator limitation. Parameter r_H represents the herbivore attack rate. This figure corresponds to Figure 4 in the main text. Where parameter combinations create stable equilibria, abundance is shown in the green color scale. Where values create stable limit cycles, abundance is shown in the sunset color scale. The switch between the two color schemes represents the Hopf bifurcation shown in Figure 3. Values which lead to system extinction are shown in white. a.) Value of H in the asymptotic behavior of the model. b.) Value of P in the asymptotic behavior of the model. $r_F = 0$; $b_F = 1.665$; $b_P = 1.695$; $d_F = 0.2$; $d_H = 0.5$; $d_P = 0.2$; $a = 0.1$, $c_{FH} = 1$, $h_H = h_P = 1$. See Table 1 in the main text for parameter and variable definition.

It is worth noting that an increase in c (decrease in pollinator visitation), does not necessarily result in a negative effect on F abundance. When the system produces a stable Equilibrium 4, there is no cost to F as c increases (Figure 4). This can be analytically verified by recalling that the parametric expression for $F^* = \frac{d_H}{c_{FH} r_H - d_H h_H}$ has no reliance on the value of c. Additionally, it is also possible to show that both P^* and H^* are inversely proportional to the value of c. However, the examples given in Figure 3a and Supplementary Figure 6b show that the effect of c on P^* is small. The effect of visitation reduction on H^* is much more pronounced (Supplementary Figure 6a).

Potential costs in oscillating populations due to reduction in pollinator visitations (reduced interaction with the mutualist) are also limited. While higher c values decrease the maximum abundance in F and P (Figure 3a, Figure 4) when the system produces sustained population oscillations, the minima of these oscillations increase (Figure 3a). This reduces the system's tendency to produce small population sizes during its cycles, thereby keeping a higher minimum population number of the two mutualistic interactors (e.g. plant and pollinator). This limits periods of exceptionally low population growth when one of the mutualists has low abundances.

Finally, we have shown that higher levels of c can cause the system to persist in a non-zero attractor despite higher levels of herbivory. Additionally, for any given value of r_H, higher levels of visitation reduction can also expand the range of mutualism growth the system can support. Another source of system failure besides increased levels of herbivory is higher growth rates of the mutualists. In the same way that high r_H can saturate the system with herbivores, high b_F and b_P can also lead to more available resources for herbivores, leading to herbivore saturation and system failure. Herbivore induced pollinator visitation decline attenuates this indirect saturation effect and expands the range of b_F and b_P

which doesn't lead to system failure. Perhaps more intuitively, sufficiently low mutualism growth rates can also lead to system failure. If growth rates of the pollinator or flowering plant are too low, the mutualism may not be able to recover herbivore induced low population numbers. Higher levels of pollinator visitation decline allow the mutualism to recover from low population numbers while reducing the growth rate when herbivores are too abundant. An example of these effects is shown in Supplementary Figure 7. Here it is clear that as c increases from 0, the range of b_F and b_P parameter space which leads to a non-zero dynamic (persistent communities) expands.

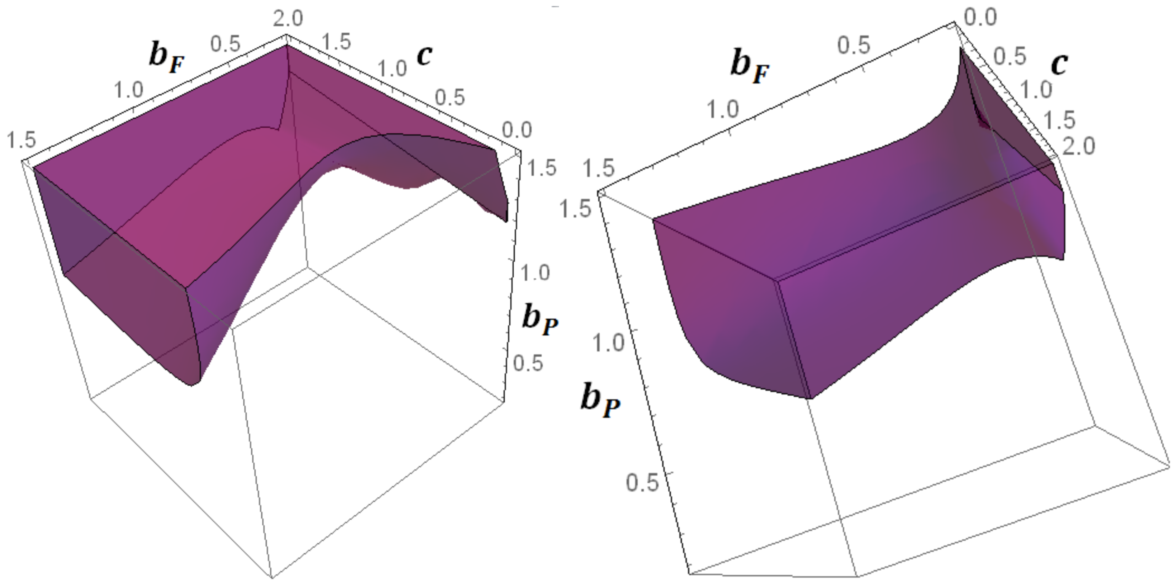

Supplementary Figure 7 | Parameter space supporting persistence.
Parameters space which supports persistence in all three populations across $\{c, b_F. b_P\}$ parameter space is shown in translucent purple. Parameter c represents the degree of herbivore-induced pollinator limitation. Parameter b_F represents the reproductive benefit of pollination to the flowering plant population. Parameter b_P represents the reproductive benefit of pollination to the pollinator population. Persistence is not distinguished between equilibria and limit cycles in this case. Simulations were run with r_H at the relatively high value of 1. Other parameters were as follows: $r = 0$, a=0.1, $d_F = 0.2,\ d_H = 0.5,\ d_P =$ 0.2, $c_{FH} = 1$, $c = 0$, $h_H = h_P = 1$. See Table 1 in the main text for parameter and variable definitions.

Supplementary Note 5 – Highly specialized mutualism with HIPL

When r_F is near zero but still positive (for some small value ϵ such that $r_F \approx \epsilon$), the model can represent a highly specialized pollination mutualism between F and P. In this case, the pollinator is dependent on the flowering plant population, but the flowering plant population is able to maintain some

average positive growth independent of pollinator P. The model produces similar rescue and stabilization dynamics compared to the obligate mutualism in the Results section (Supplementary Figure 8).

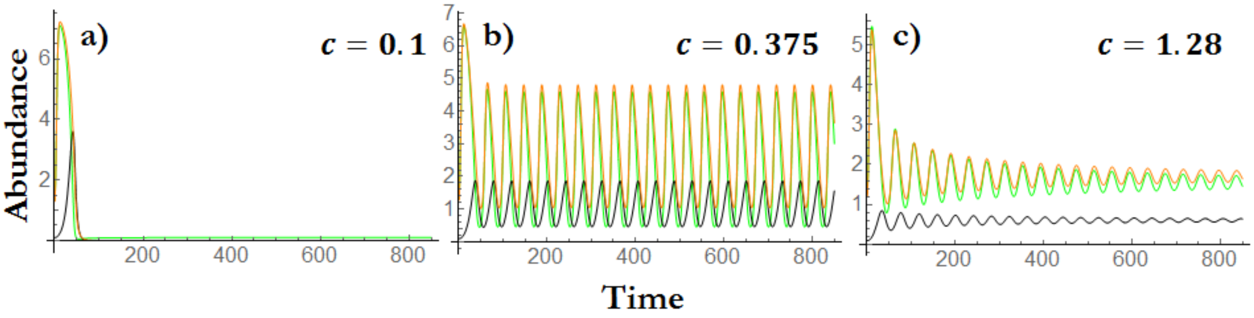

Supplementary Figure 8 | HIPL rescue effect in specialized mutualism

Example of similar rescue and stabilization dynamic driven by HIPL with higher c values in a highly specialized system where r_F is slightly greater than 0 (i.e. $r_F = \epsilon$ such that $\epsilon > 0$). In this case $r_F = 0.21$. a) System failure with low influence of HIPL, $c = 0.1$. b) Past the rescue point and establishment of sustained oscillation with more influence from HIPL ($c = 0.375$). c) Oscillations dampen and approach stable equilibrium with higher c values ($c = 1.28$). All other parameters: $r_F = 0.21$; $r_H = 0.71$; $b_f = 0.83$; $b_p = 1.08$; $c_{FH} = 0.58$; $h_H = 1$; $h_P = 1$; $d_F = 0.2$; $d_H = 0.25$; $d_P = 0.2$; $\alpha = 0.1$.

Supplementary Note 6 – Approximating the Volume of the Basin of Attraction in Obligate Model

While c values greater than 0 can induce system persistence, both rates of herbivory (r_H) and pollinator aversion to herbivory (c) have significant effects on the volume of the basin of attraction of non-zero attractors (Supplementary Figure 9). In other words, non-zero c values can create the potential for system rescue, higher values of c reduce the amount of initial conditions which move toward non-zero attractors. Recall that non-zero attractors in this case are the attractors (equilibria & limit cycles) which allow for system persistence instead of extinction (the 0-equilibrium). The basin of attraction for an attractor is the set of initial conditions in phase space that will eventually be iterated into the attractor over time. When the basin of attraction of the non-zero attractor is small, there are more initial conditions which will push the system into the 0-equilibrium, leading to extinction. When the basin of attraction of the non-zero attractors is larger, there are more initial conditions which will push the system into the non-zero attractor and the community will persist. For further description, see Strogatz 1994[6] and Supplementary Figure 4.

We determined the approximate volume of the basin of attraction in phase space for both Equilibrium 4 and limit cycles (sustained oscillations) across $\{c, r_H\}$ parameter space. There is no analytical method to study the size of the basin of attraction so it must be investigated through numerical

simulations. Through an extensive numerical survey of 2.7 $\times 10^7$ initial conditions in phase space simulated across 2800 parameter combinations we compiled a 7.56$\times 10^{10}$ point data set which develops a full understanding of how the size of the basin of attraction changes with different values of c and r_H. Stable equilibria were found using standard Jacobian stability analysis on Equilibrium 4. There is no set method to analytically determine the existence of limit cycles. Therefore, limit cycles were verified using numerical means. We determined an approximate volume of the basin of attraction in phase space for both Equilibrium 4 and limit cycles (sustained oscillations) across $\{c, r_H\}$ parameter space (Supplementary Figure 9).

Reduction in pollinator visitation (higher c) causes a sharp initial decrease in the volume of the basin, but then begins to have a smaller effect. Whereas the effect of increased rates of herbivory (r_H), cause a more consistently steep decline in basin volume over less parameter space. This result suggests that while visitation reduction can facilitate 3-variable coexistence at r_H levels that would otherwise cause system extinction, the basins of attraction for the non-zero attractors are smaller with higher r_H. In other words, the potential for persistence offered by pollinator visitation reduction comes with the caveat of susceptibility to perturbations. There is also a clear continuation of the non-zero attractors' basin volume across the bifurcation from stable equilibrium to limit cycles. With this result, we can say that the prominent dynamic (equilibria or oscillations) in the model does not affect the basin volume of the non-zero attractor. Instead, it is the parameter values themselves which lead to changes in volume.

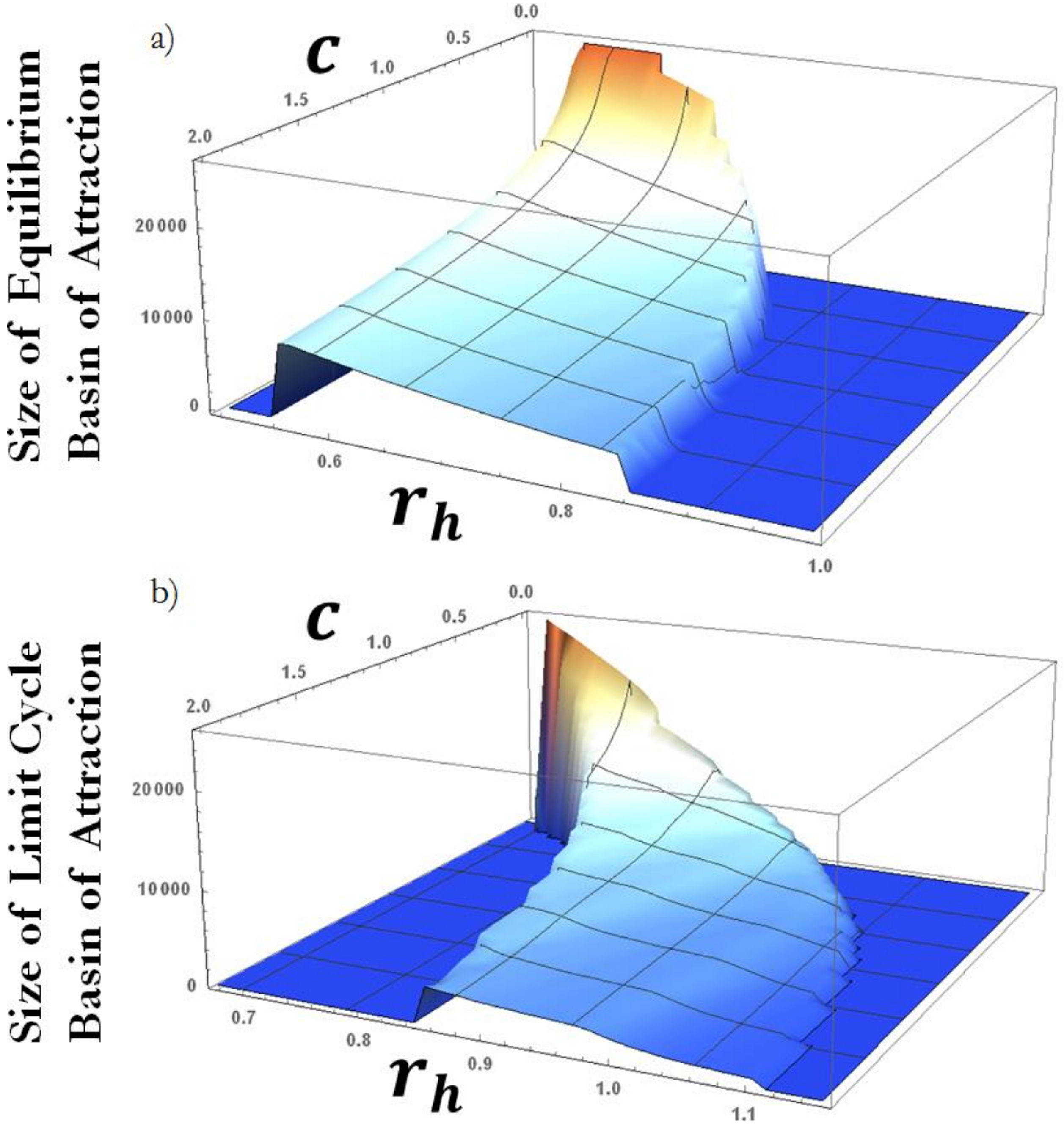

Supplementary Figure 9 | Size of Equilibrium 4's Basin of Attraction across parameter space. Numerical approximation of the phase space unit volume of basins of attraction as a function of r_h (the rate of herbivory) and c (the degree of pollinator visitation reduction due to herbivory) for a) the stable equilibria and b) the stable limit cycles. The volume was approximated through testing asymptotic system behavior across all initial conditions from 0 to 15 for each variable. This was repeated across different combinations of c and r_h values. The number of initial conditions which result in equilibrium behavior is a suitable approximation of the basin of attraction's unit area in phase space. $r_F = 0$; $b_F = 1.665$; $b_P = 1.695$; $d_F = 0.2$; $d_H = 0.5$; $d_P = 0.2$; $h_F = 1$; $h_P = 1$; $c_{FH} = 1$; $\alpha = 0.1$.

Supplementary Note 7 - Facultative mutualism with HIPL

Setting r_F substantially greater than 0 (r_F greater than some small value ϵ, $r_F > \epsilon > 0$) leads to complications in the effects of visitation reduction. As shown in the main paper, non-zero values of r_F can lead to a population crash for the pollinator. The potential for a high growth rate of one facultative mutualist to crash its obligate partner has to do with the growth of the antagonist (in this case the herbivore). When the system is stabilized in equilibrium by visitation reduction and r_H is low, with an overall growth rate of $(r_H - d_H)$ of roughly 0.3, increasing r_F can keep the system in equilibrium while pushing $P^* \to 0$ (Supplementary Figure 10 and Figure 5). As was shown in the main paper, inducing oscillations and increasing their amplitude by further increasing r_F can create windows of time where r_H is low and P is allowed time to grow.

Additionally, the 2-parameter bifurcation diagram in Supplementary Figure 10 shows a similar effect is possible when r_H is increased. As r_H is increased, we can see an initial drop in equilibrium values of P^* and an eventual rebound in peak values after the induction of limit cycles. The reasoning here is similar to when pollinator populations are saved by higher r_F. The fast growth of herbivore populations will induce limit cycle behavior in the plant-herbivore Lotka-Volterra consumer-resource system. This will cause high peaks in herbivore abundance that will lead to prolonged troughs in flowering plant populations. This will consequently drop the population of herbivore long enough for the small populations of pollinators to begin to grow in the interim. The result described here is similar to that described in Figure 5c.

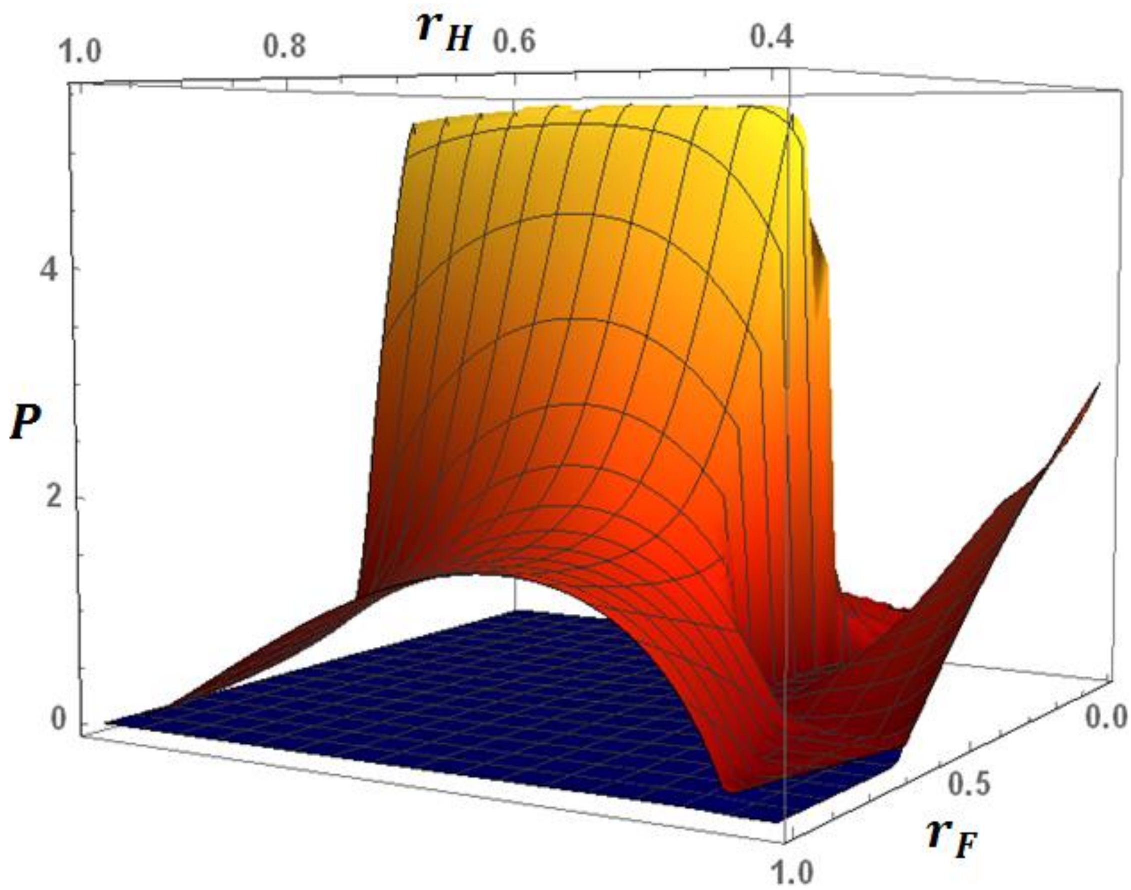

Supplementary Figure 10 | 2-D bifurcation for P.

A two dimensional bifurcation plot for P (pollinator population) across different parameter values of r_F and r_H (intrinsic growth rate of flowering plant population and herbivore attack rate respectively). Combinations which create stable equilibria have just one P value plotted whereas combinations which create limit cycles are plotted with both a P minimum and P maximum. Values are plotted with fitted curves to show contours. Maximum limit cycle values and equilibria values are shown in sunset colors. Minimum limit cycle values are shown in dark blue. Areas with no minimum value shown (right side of figure) are equilibria. At both low and high values of $r_H$an increased intrinsic growth rate of the flowering plant (higher r_F) can actually lead to reductions in pollinator abundance. $b_F = 1.04;\ b_P = 0.85;\ c = 1.2;\ d_F = 0.2; d_H = 0.302; d_P = 0.2; a = 0.1; h_F = 1; h_P = 1; c_{FH} = 1$. See Table 1 in the main text for parameter and variable definitions.

This surprising benefit to the pollinator of higher herbivore growth can be hindered in two ways. First, and most intuitively, if r_H is too high, the troughs in H abundance are short and P is not afforded as much time to grow. This results in a decline in peak P abundance (Supplementary Figure 10). Second, increased intrinsic growth of F (i.e. higher r_F) can speed the growth of H populations in troughs and limit

time available for P population growth. All of these various conditions and tradeoffs for pollinator growth create a complicated condition for pollinator persistence that depends on visitation reduction, growth rate of the herbivore, and the intrinsic growth rate of the flowering plant (values of c, r_H, r_F respectively).

In order to investigate the effect of each of these key parameters of the pollinator population, we compiled a large numerical analysis of P abundance in asymptotic model behavior (Supplementary Figure 11). Equation 1 was simulated 76880 times across values of c, r_H, and r_F and the depending on the dynamic of the model (stable equilibrium or limit cycles) the equilibrium value or peak limit cycle value of P was recorded. These values of P are presented as a color gradient. The data shown in Supplementary Figure 11 only represents situations where the flowering plant has a high enough r_F that it can survive without pollination.

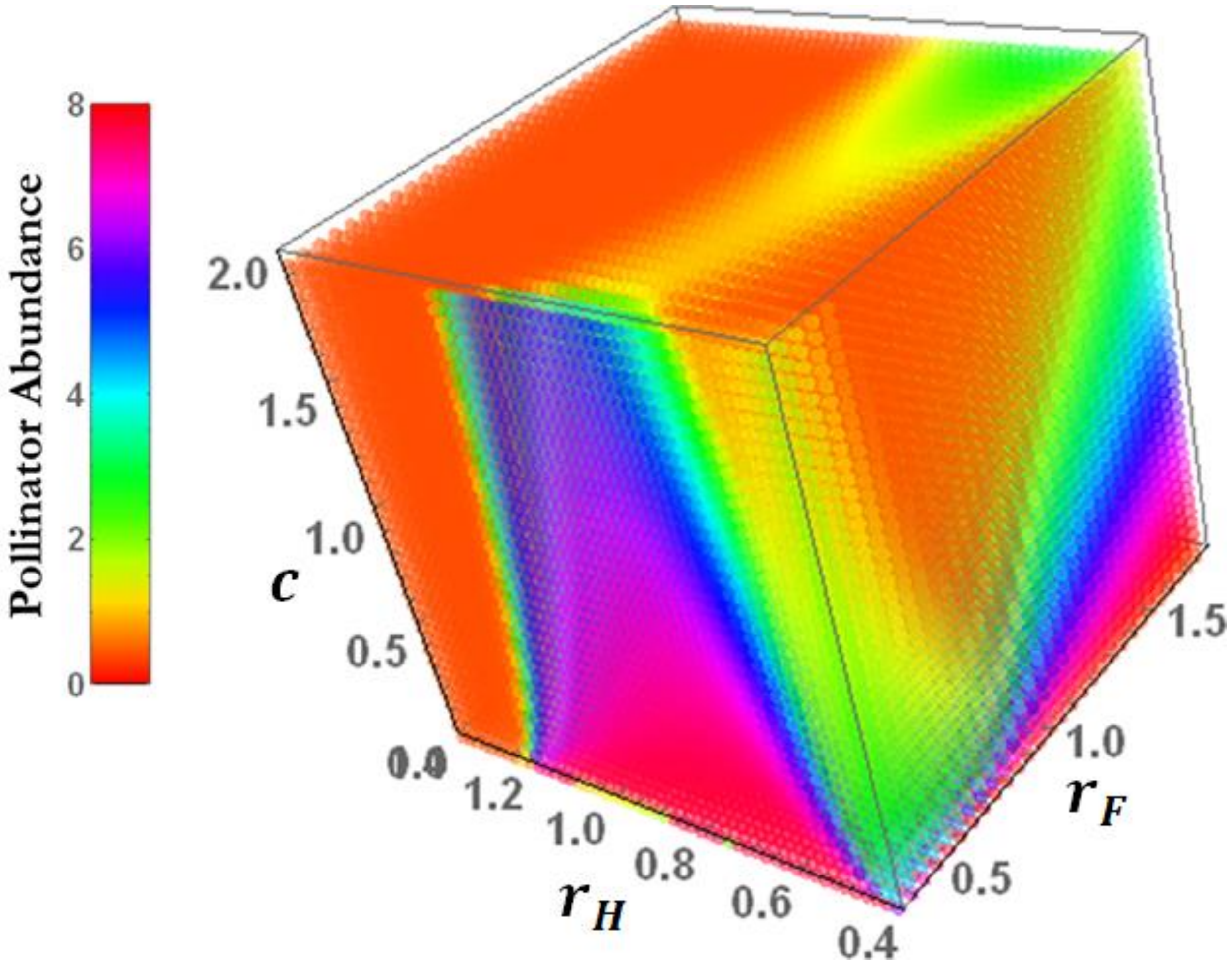

Supplementary Figure 11 | Pollinator abundance across parameter space.

The equilibrium value or peak limit cycle value of P (pollinator population) at separate values of c, r_H, r_F. Parameter c represents the degree of herbivore-induced pollinator limitation. Parameter r_H represents the

attack rate of the herbivore population. Parameter r_F represents the intrinsic reproductive rate of the flowering plant population. Colors represent either the equilibrium value of P or the value it takes at the maximum of oscillations in limit cycles. $b_F = 1.04, b_P = 0.85, a = 0.1, d_F = 0.2, d_H = 0.302, d_P = 0.2$; $h_F = 1$; $h_P = 1$; $c_{FH} = 1$. See Table 1 in the main text for parameter and variable definitions.

Supplementary Note 8 – HIPL driven rescue effect with Type I Functional Response

While we argue that the Type II functional response has the most support in the available data, it is possible that other pollination systems may support a different functional form. Therefore, there is merit in investigating the consistency of the rescue effect provided through HIPL when using other functional responses in the model. In other words, here we will test the potential for the rescue effect with the Type I, Type III, Mixed Saturating, and Concave functional responses. Analysis shows that the rescue effect can be readily replicated across all functional response forms, with only the Concave response showing a noticeable reduction in the range of parameter space supporting community persistence. The Type I functional response for HIPL is the linear equation, $v(c, H) = 1 - c * H$. We can incorporate this form of $v(c, H)$ into the model using the Piecewise function:

$$v(c, H) = \begin{cases} 1 - c * H & when\ 1 > c * H \\ 0 & when\ 1 \leq c * H \end{cases} \tag{S10}$$

The Piecewise formulation stops $v(c, H)$ from becoming negative at any time in simulations. This formulation means that $v(c, H)$ decreases linearly with increased herbivore abundance (H) until it reaches 0. The value of $v(c, H)$ then remains at 0 when $H \geq 1/c$. As in the main paper, the interaction rate of pollinators and flowering plants is assumed to be 1 when herbivore abundance and damage is zero. With this instantiation of the model, the linear Type I HIPL functional response can still produce the rescue effect. It is possible to create similar bifurcation heatmaps as shown in Figure 4 in the main paper to illustrate this result (Supplementary Figure 12).

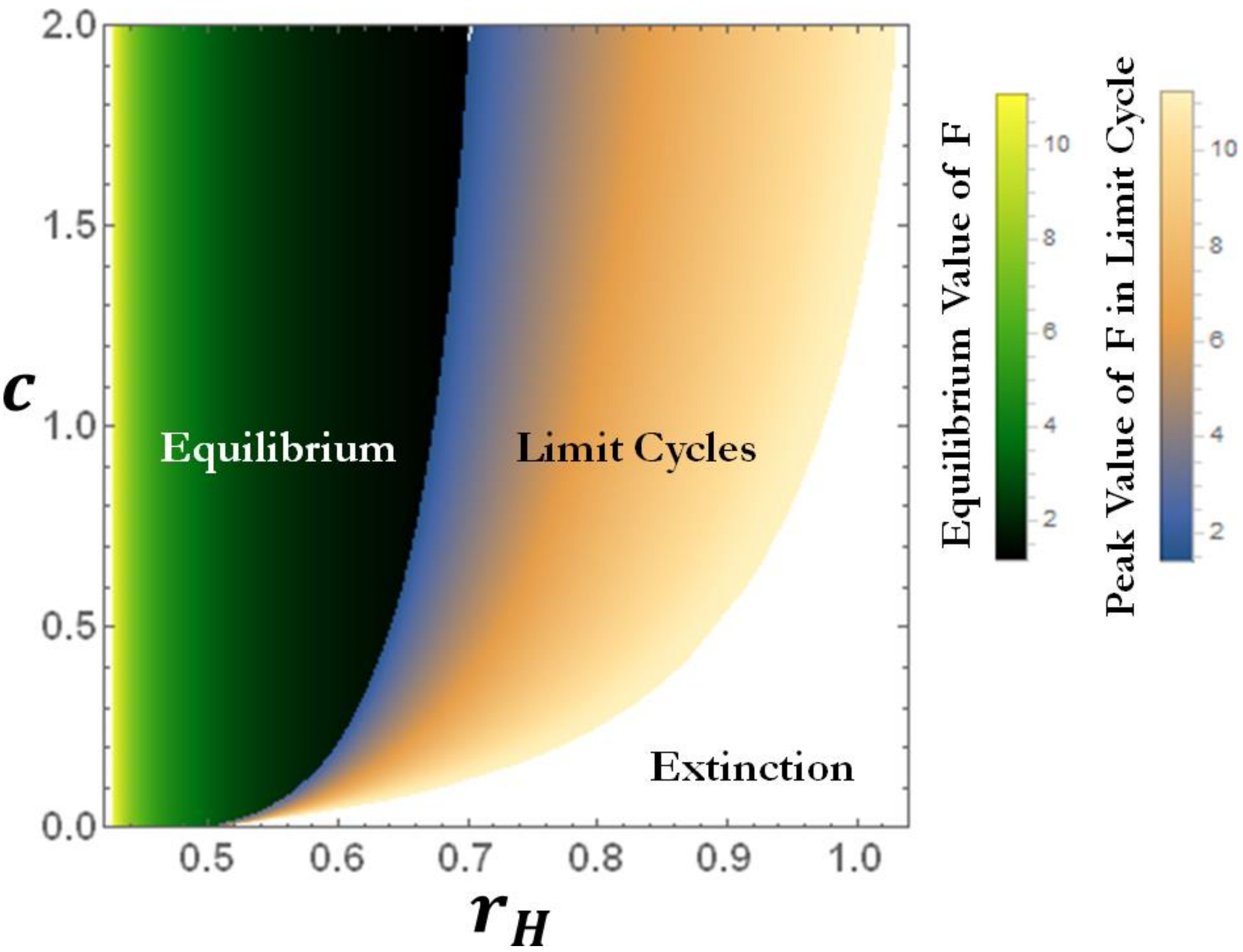

Supplementary Figure 12 | 2-D bifurcation heatmaps with TYPE I HIPL.

A two-dimensional bifurcation heatmap showing the abundance of F (flowering plant) in the asymptotic behavior of the model using a TYPE I functional response for HIPL. Different asymptotic behaviors of the model are shown as different colors across the $\{r_H, c\}$ parameter space. Where parameter combinations create stable equilibria, F abundance is shown in the green color scale. Where values create stable limit cycles, F abundance is shown in the sunset color scale. Areas in white represent herbivore driven local extinction. $r_F = 0$; $b_F = 1.265$; $b_P = 1.4$; $c_{FH} = 0.7$; $d_F = 0.2$; $d_H = 0.25$; $d_P = 0.2$; $h_F = h_P = 1.1$; $\alpha = 0.1$.

Supplementary Note 9 – HIPL driven rescue effect with Type III Functional Response

Similar to the Type I and Type II functional response, the Type III form of HIPL was also found to produce the rescue effect. In this case, $v(c, H) = \frac{1}{1+cH^2}$. Again we present the results in the bifurcation heatmap figure (similar to Fig 3 in the main paper). The Type III functional response can allow for the rescue effect over similarly large subset of the parameter space (Supplementary Figure 13).

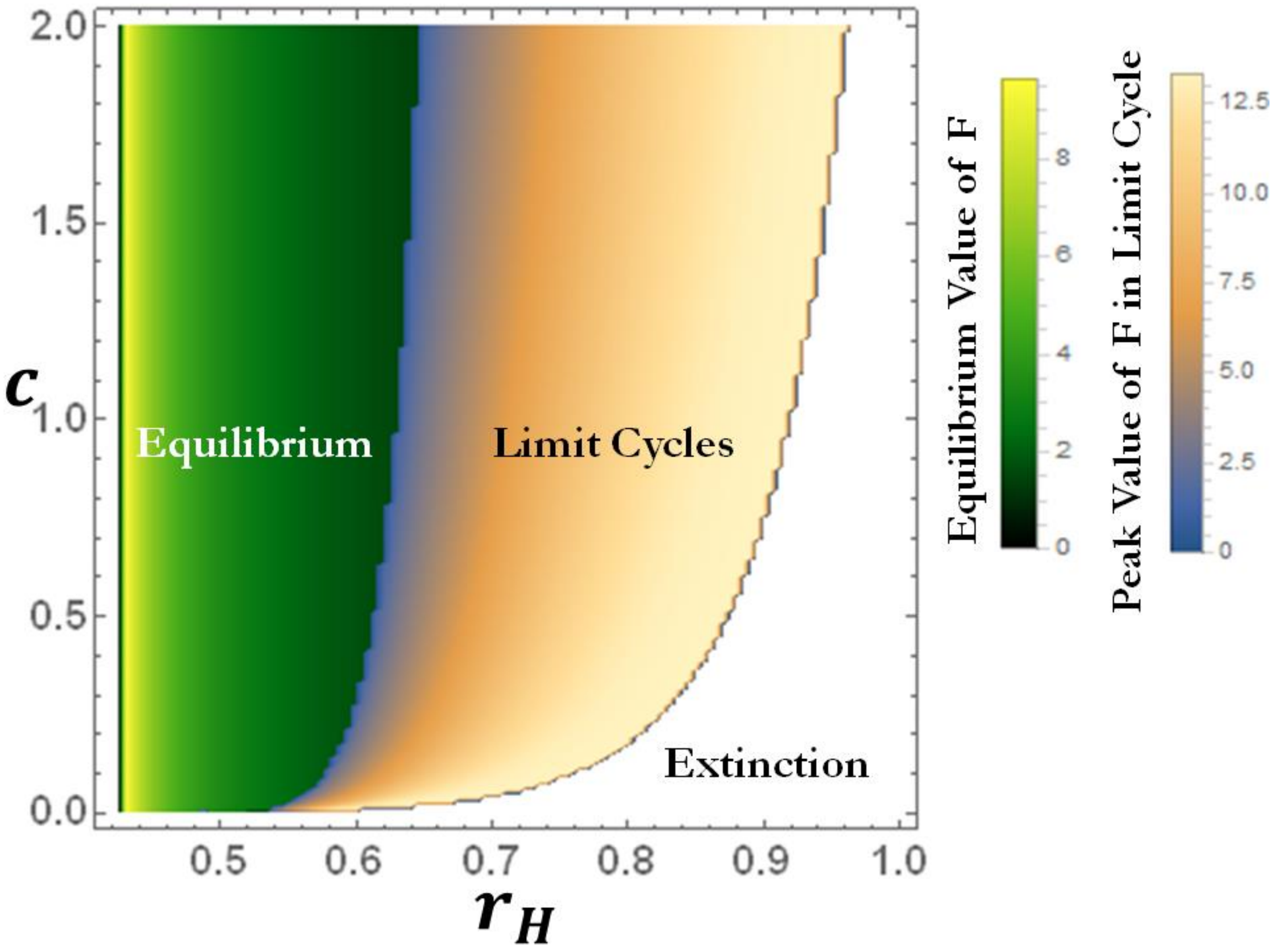

Supplementary Figure 13 | 2-D bifurcation heatmaps withTYPE III HIPL.

A two-dimensional bifurcation heatmap showing the abundance of F (flowering plant) in the asymptotic behavior of the model using a TYPE III functional response for HIPL. Different asymptotic behaviors of the model are shown as different colors across the $\{r_H, c\}$ parameter space. Where parameter combinations create stable equilibria, F abundance is shown in the green color scale. Where values create stable limit cycles, F abundance is shown in the sunset color scale. Areas in white represent herbivore driven local extinction. $r_F = 0$; $b_F = 1.465$; $b_P = 1.615$; $c_{FH} = 0.7$; $d_F = 0.2$; $d_H = 0.25$; $d_P = 0.2$; $h_F = h_P = 1.1$; $\alpha = 0.1$.

Supplementary Note 10 – HIPL driven rescue effect with Mixed Saturating Functional Response

The Type III functional response is actually a subset/subcase of the Mixed Saturating form. From a modeling standpoint (both statistical and dynamic), the Mixed Saturating Case is a more complicated case because there are three parameters to test ($\{r_H, c, b\}$). Regardless, the Mixed Saturating form can produce the rescue effect result described in the main paper, but the details are more involved. The Type I, II, III functional responses only had one parameter per function (c), so it was possible to make the 2-D

bifurcation heatmaps. In this case, there is more than one parameter for the mixed saturating functional response (parameter c and parameter b):

$$v(c, b, H) = \frac{1}{1+cH^b} \tag{S11}$$

Therefore, the previous 2-D $\{r_H, c\}$ bifurcation heatmaps do not show all the details and we will need to show multiple figures to describe the full dynamics. In this model we are actively changing the values of b for the first time, so we initially parse through values of b to test their comparative effects. The values of b will vary from 1 to 3 allowing us to compare dynamics of a Type II response ($b = 1$) with a Type III ($b = 2$) and the Mixed Saturating case (b generally greater than 1). First, testing the effect of b on a lower interacting system reveals that higher values of b do not restrict the range of community persistence (Supplementary Figure 14a). In fact, on the lower values of c, it appears higher b values allow for community persistence. Testing a more interactive system (Supplementary Figure 14b) offers clearer support for this idea.

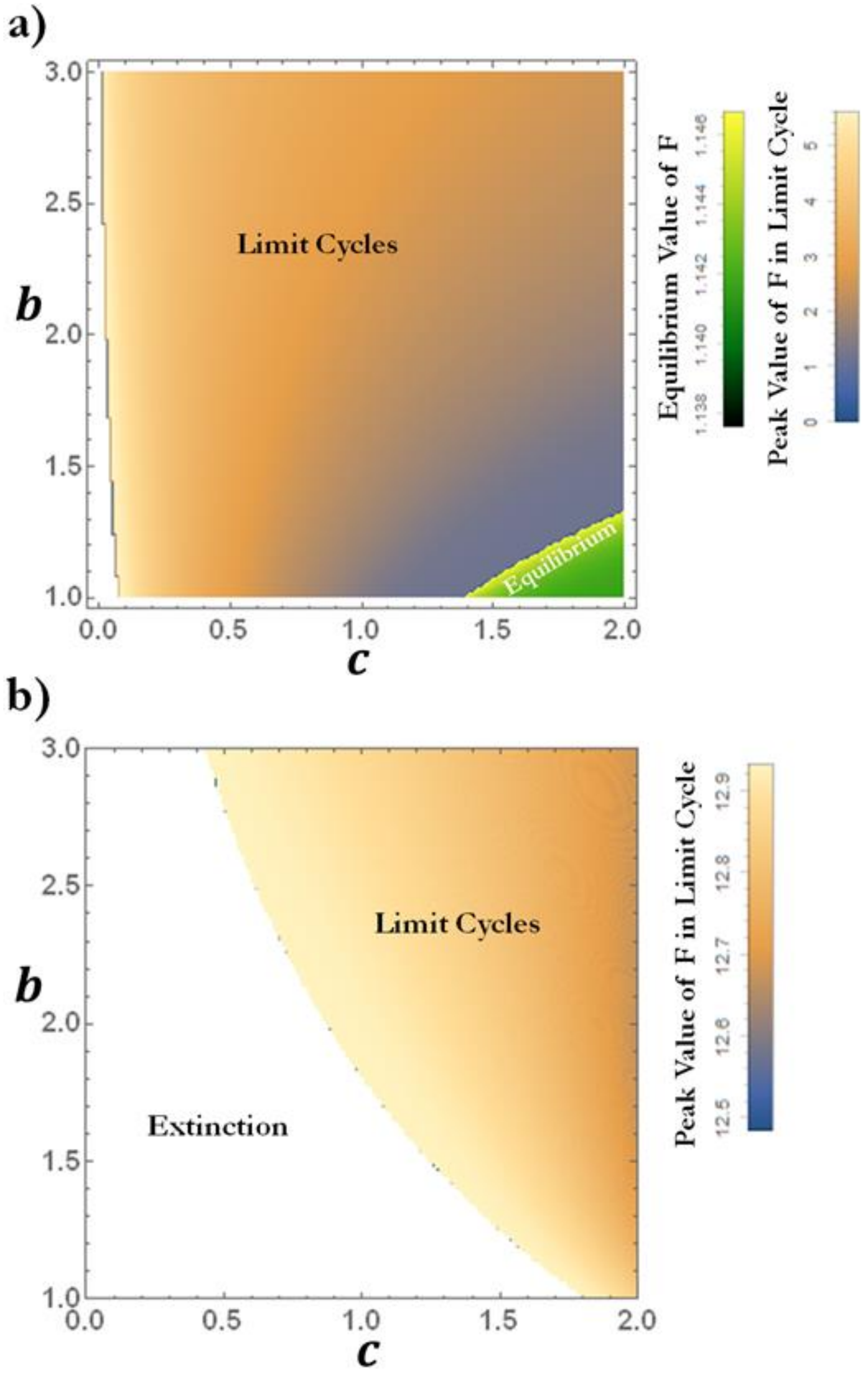

Supplementary Figure 14 | 2-D bifurcation heatmaps with Mixed Saturating HIPL.

a) Two-dimensional bifurcation heatmaps showing the abundance of F (flowering plant) in the asymptotic behavior of the model using a Mixed Saturating functional response for HIPL. Different asymptotic behaviors of the model are shown as different colors across the $\{b, c\}$ parameter space. Where parameter combinations create stable equilibria, F abundance is shown in the green color scale. Where values create stable limit cycles, F abundance is shown in the sunset color scale. Areas in white represent herbivore driven local extinction. a) $b_F = b_P = 0.78$, $r_H = 0.67$, $c_{FH} = 0.7$; $d_F = 0.2$; $d_H = 0.25$; $d_P = 0.2$; $h_F = h_P = 1.1$; $\alpha = 0.1$. b) $b_F = 1.45$, $b_P = 1.55$, $r_H = 0.91$, $c_{FH} = 0.7$; $d_F = 0.2$; $d_H = 0.25$; $d_P = 0.2$; $h_F = h_P = 1.1$; $\alpha = 0.1$.

To more fully understand the role of parameter b in model dynamics and the rescue effect, we expanded the parameter sweep to include r_H so that persistence could be measured across over 453,000 parameter combinations in $\{c, b, r_H\}$ parameter space. The parameter sweep done to construct this graph was done with r_H values from 0.45 to 1.0 with 0.01 steps, c values from 0.0 to 2.0 with 0.02 steps, and b values from 1 to 3 with 0.02 steps. Upon completion of the analysis, we found that increased values of b expand the range of the HIPL derived rescue effect in $\{r_H, c\}$ parameter space (Supplementary Figure 15). As indicated in Supplementary Figure 14b, this expansion largely results from the reduction in the value of c required to sufficiently control the herbivore population prompting the rescue effect.

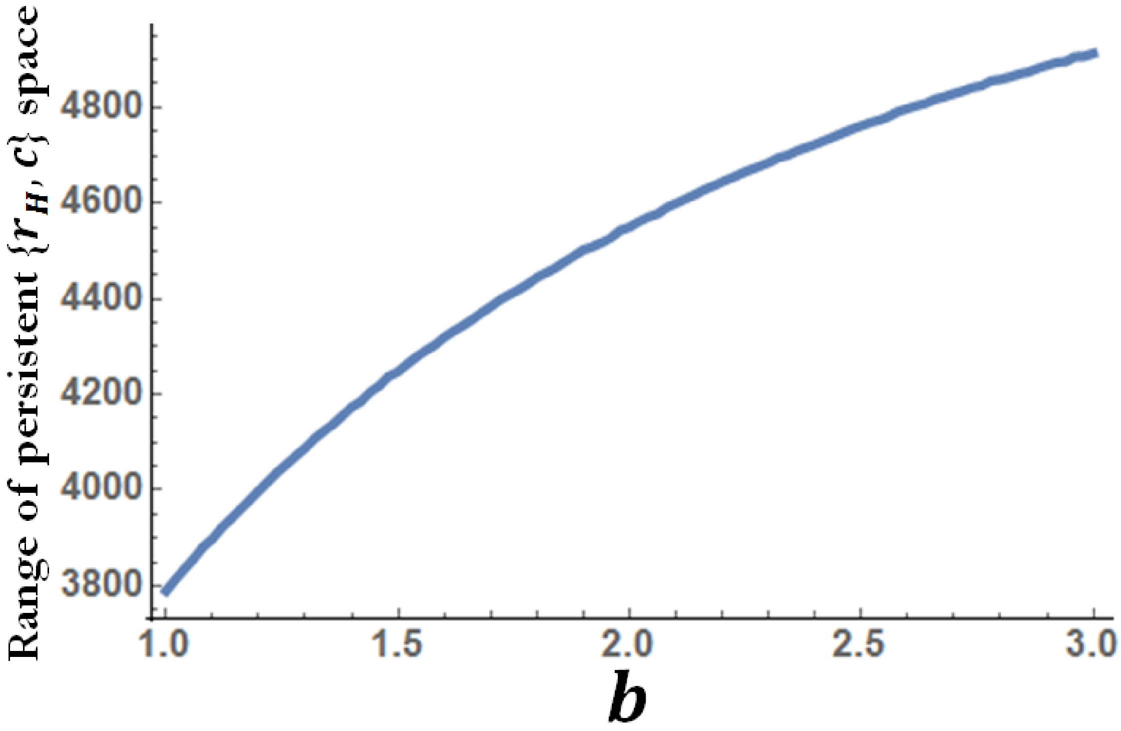

Supplementary Figure 15 | Change in persistent parameter space across values of b.

Measurements of the number of $\{r_H, c\}$ parameter combinations across a range of b values where the HIPL rescue effect enables persistent communities. The y-axis in the case is the actual count of distinct parameter combinations which HIPL supports persistent communities. This shows the range of the rescue effect increases with higher values of b. Parameter values are $b_F = 1.45, b_P = 1.55, c_{FH} = 0.7,\ d_F = 0.1, d_P = 0.1, d_H = 0.1, h = 1.1,\ \alpha = 0.1$.

Higher values of b not only reduce the level of c required for persistence, they also slightly increase the level of herbivore attack rate (i.e. higher r_H) that the system can withstand before local extinction (Supplementary Figure 16). The heatmap in Supplementary Figure 16 does not show asymptotic value of the flowering plant population as it does in other heatmaps. Instead it shows the lowest value of c (lowest level of HIPL) that the system could withstand and still persist. Intuitively, lower values of r_H require lower values of c for the rescue effect. Higher values of b also can also decrease the minimum value of c needed to support community persistence. This is shown in two color schemes in Supplementary Figure 16 to make the point clearer.

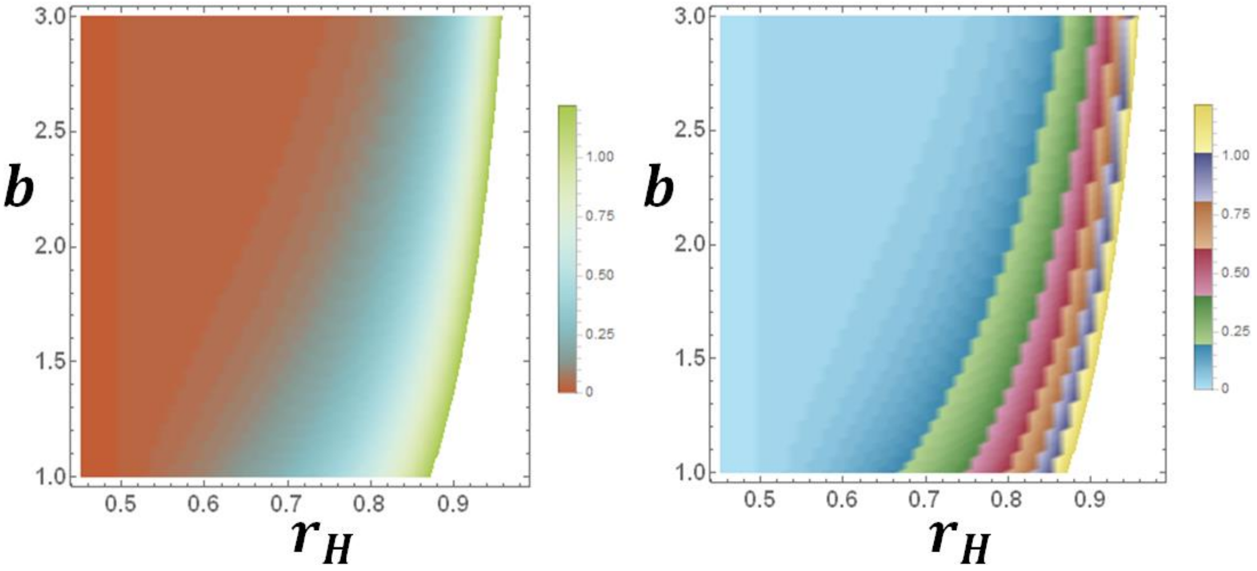

Supplementary Figure 16 | Minimum value of c required for community persistence. Heatmap showing the minimum value of c required to support system persistence through the rescue effect of HIPL across the range of all tested values of r_H and b. This minimum value of c is shown in the colors of each graph explained by the color legend to the right of each figure. The same figure is shown with two different color schemes to represent the minimum required c value in an easily visible manner. Parameter values are $b_F = 1.45, b_P = 1.55, c_{FH} = 0.7,\ d_F = 0.1, d_P = 0.1, d_H = 0.1, h = 1.1, \alpha = 0.1$.

The cause of this increase in the range of community persistence across parameter space results from the fact that higher b values (i.e. $b > 1$) do two things to the shape of the $v(c, H)$ function. First, it

creates a delay in the immediate effect of HIPL, such that higher herbivore abundance is required to see a decrease in pollinator visitation ($v(c, b, H)$). Second, once the herbivores are abundant, higher b values make the resulting decrease in pollinator visitation progressively steeper, such that the decline in pollinator visitation is quite rapid. While the first effect would seem detrimental, when coupled with the second effect, it can actually be beneficial. A less immediate decline in $v(c, b, H)$ at low herbivore abundance can actually help the pollinator and plant populations rebound during troughs in the population trajectory because low herbivore abundance won't impede pollination. This effect by itself would then fail to control herbivore populations as the populations rebounded, but the concurrent steep decline in pollination once herbivore abundance become sufficiently high (the second effect above), helps regain the indirect control of the herbivore population growth through greater reductions in v (Supplementary Figure 17).

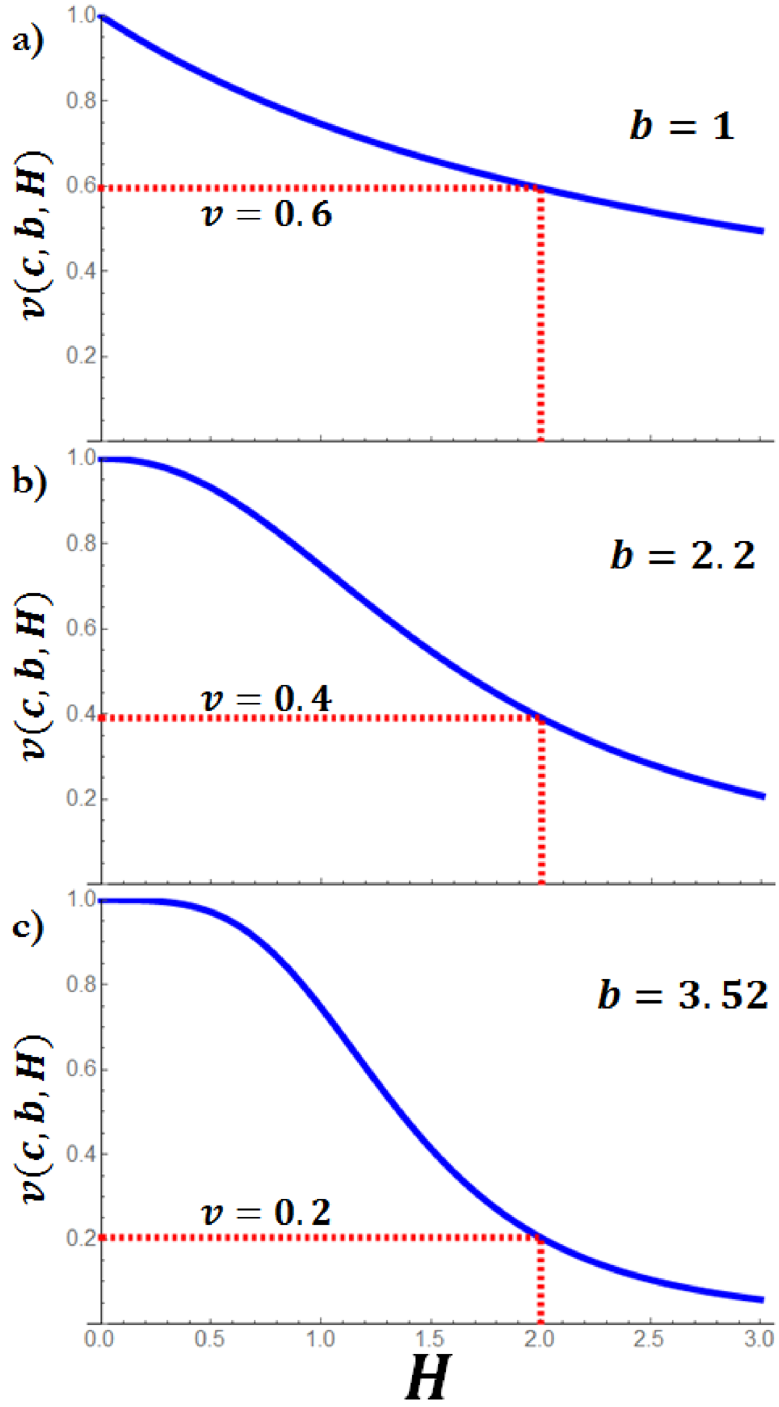

Supplementary Figure 17 | Changes in the shape v for different values of b.

The resultant value of the pollination visitation parameter $v(c, b, H)$ (shown in blue) across herbivore abundance (H) at different levels of the parameter b when using the Mixed Saturating functional response form: a) $b = 1$, b) $b = 2.2$, c) $b = 3.52$. The red dashed line shows the value of $v(c, b, H)$ when the herbivore abundance (H) equals 2.0. Here, $c = 0.34$. As the value of b increases, the shape of the

function changes such that the eventual decrease in $v(c, b, H)$ becomes very steep. Therefore, $v(0.34, b, 2)$ decreases resulting in more HIPL and lower pollination rates.

Supplementary Note 11 – HIPL driven rescue effect with Concave functional response

Various numerical simulations show that it is possible to recreate the rescue effect with the Concave Functional Response (Supplementary Figure 18). However, the Concave functional response generally created the smallest parameter space in which the rescue effect could be found. By creating the longest delays in declining the pollinator visitation rate (v), the Concave functional response can significantly hinder any possible direct control of the herbivore population through HIPL. The concave model is the least supported direct curve fit we attempted, so we claim that the only functional response type that noticeable reduces the range of the rescue effect in the model does not seem well supported. With this we can claim that the main results presented in the paper are robust to most functional responses types.

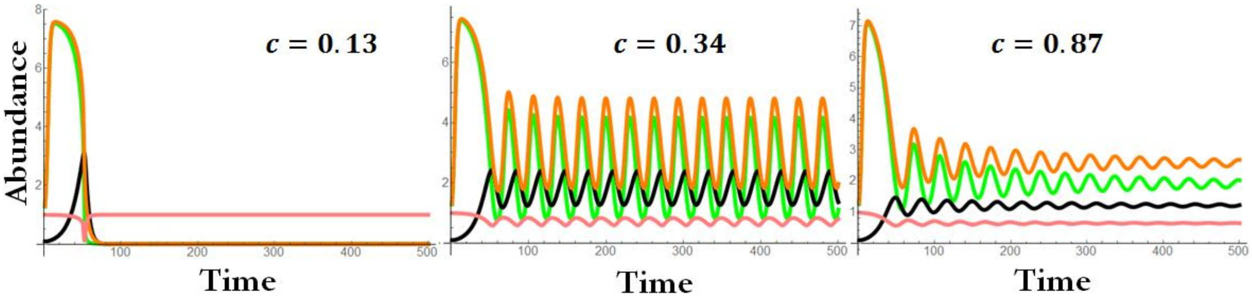

Supplementary Figure 18 | Rescue effect of HIPL with Concave functional response.
Three time series showing the rescue effect using a Concave functional form of HIPL. Parameter values: $r_H = 0.58,\ b_F = 1.095,\ b_P = 1.095,\ c_{FH} = 0.7, h = 1.1,\ c = 0.13,\ 0.338,\ 0.868, \alpha = 0.1, d_F = 0.1, d_P = 0.1, d_H = 0.1$,. The green line, orange line, and black line represent the flowering plant, pollinator, and herbivore respectively. The pink line is the value of the $v(c, H)$ function as a response to the herbivore abundance.

References:

1.) Kessler A, Halitschke R, Poveda K. Herbivory-mediated pollinator limitation: negative impacts of induced volatiles on plant–pollinator interactions. Ecology 92(9): 1769–1780 (2011).

2.) Barber N, et al. Herbivory reduces plant interactions with above- and belowground antagonists and mutualists. Ecology 93(7): 1560-1570 (2012).

3.) Krupnick GA, Weis AE, Campbell DR. The consequences of floral herbivory for pollinator service to Isomeris arborea. Ecology 80:125–134 (1999).

4.) Adler LS & Irwin RE. Ecological costs and benefits of defenses in nectar Ecology. 86(11): 2968–2978 (2005).

5.) Kessler A & Halitschke R. Testing the potential for conflicting selection on floral chemical traits by pollinators and herbivores: predictions and case study. Functional Ecology 23: 901–912 (2009).

6.) Strogatz SH. Nonlinear Dynamics and Chaos: with Applications to Physics, Biology, Chemistry and Engineering. Westview Press, 11 Cambridge Center, Cambridge, MA 02142 (1994).
