# Supplementary material for: Functional reduction in pollination through herbivore-induced pollinator limitation and its potential in mutualist communities
Source: Nat Commun. 2017 Dec 11;8:2031. doi: 10.1038/s41467-017-02072-4 (PMC5725495; doi:10.1038/s41467-017-02072-4)
Supplement: Supplementary file 3 — Description of Additional Supplementary Files [file 41467_2017_2072_MOESM3_ESM.pdf]

Description of Additional Supplementary Files

File Name: Supplementary Data 1
Description: Field data used to find functional form of HIPL and to compile Figures 1 and 2.
